# Postoperative outcomes in patients with gastric cancer undergoing prehabilitation in a high-complexity referral centre

**DOI:** 10.1186/s12957-026-04310-w

**Published:** 2026-05-04

**Authors:** Oscar Julian Suescun Fajardo, Sameer Torres Gaviria, Mauricio Chona Chona, Lina López Basto, Jorge Medina Parra, Ricardo Merchán Chaverra

**Affiliations:** 1 Departamento de Cirugía General, Universidad del Rosario, Bogotá, Colombia; 2 Departamento de Epidemiología Clínica, Fundación Universitaria de Ciencias de la Salud FUCS, Bogotá, Colombia; 3 Departamento de Cirugía General,Grupo Keralty, Clínica Universitaria Colombia, Bogotá, Colombia; 4 Vicepresidencia de innovación y desarrollo científico, Clínica Infantil Santa María del Lago,Clínica Reina Sofía Pediátrica y Mujer, Clínicas Colsanitas, Grupo Keralty, Clínica Universitaria Colombia, Bogotá, Colombia; 5 Grupo de Investigación en Nutrición Clínica y Rehabilitación, Clínicas Colsanitas, Grupo Keralty, Fundación Universitaria Sanitas, Bogotá, Colombia

**Keywords:** Stomach neoplasms, Preoperative exercise, Nutritional status, Infection, Malnutrition, Gastrectomy

## Abstract

**Background:**

Gastric cancer remains one of the most frequent oncological diseases, often associated with a high rate of postoperative complications. Prehabilitation has shown benefits in other surgical settings, although its role in gastric cancer patients remains under investigation.

**Objective:**

To describe the postoperative outcomes of patients with gastric cancer who underwent a prehabilitation programme in a high-complexity referral centre in Bogotá, Colombia.

**Methods:**

A descriptive observational retrospective cohort study was conducted at Clínica Universitaria Colombia between January 2021 and December 2023. Patients aged 18–80 years with a confirmed diagnosis of gastric cancer who underwent surgical treatment were included.

**Results:**

A total of 140 patients with gastric cancer received prehabilitation. The mean age was 60.9 years, and 60% were male. Postoperative complications occurred in 23.6% of patients, with surgical site infection being the most frequent (17.1%). Admission to the intensive care unit was required in 7.1%, and overall mortality was 4.3%. In bivariate analysis, malnourished patients presented higher rates of total complications, surgical site infection, and ICU admission.

**Conclusions:**

Malnutrition is consistently associated with worse postoperative outcomes. Therefore, prehabilitation plays a crucial role in improving nutritional and functional parameters that directly influence the recovery and prognosis of patients with gastric cancer.

## Introduction

Gastric cancer ranks as the fifth most commonly diagnosed malignancy worldwide and the fourth leading cause of cancer-related mortality, according to the most recent GLOBOCAN estimates (1). Surgical resection remains the cornerstone of treatment; however, radical surgery carries inherent risks and a substantial burden of morbidity, with a direct impact on short- and medium-term survival (2). Consequently, over recent decades, one of the main challenges for surgeons has been to identify and understand the factors that influence postoperative outcomes.

The *Enhanced Recovery After Surgery* (ERAS) Society has proposed multimodal, evidence-based protocols designed to optimize perioperative care, attenuate the inflammatory response induced by surgery, and thereby improve clinical outcomes (3, 4). In gastric cancer, the implementation of ERAS-based strategies has demonstrated significant benefits, including fewer postoperative complications and faster functional recovery (5, 6).

More recently, an emerging concept has gained relevance within perioperative care: prehabilitation. Traditionally, surgical care has focused on postoperative rehabilitation; however, prehabilitation aims to optimize patients’ physical, nutritional, and functional status before surgery, promoting faster and more effective recovery (7). Both preoperative functional capacity and nutritional status are strong predictors of postoperative outcomes, and their optimisation has been shown to enhance recovery and reduce complications (8, 9).

Evidence supporting prehabilitation programmes continues to expand, with documented benefits across both oncological and non-oncological populations, including colorectal (10), thoracic (11), vascular (12), gynaecologic-oncologic (13), and elective cardiac surgery (14), among others (15). In gastric cancer, growing evidence suggests that prehabilitation is a safe and effective intervention associated with lower rates of infectious and non-infectious complications and shorter hospital stays. These findings were corroborated in the main analysis of the SUPREMO study (16, 17, 18, 19, 20).

Therefore, the objective of the present study was to describe the clinical outcomes of patients with gastric cancer who completed a comprehensive prehabilitation programme prior to surgery in a high-complexity referral centre.

## Methods

### Study design

A retrospective cohort study was conducted at a quaternary-care referral centre in Bogotá, Colombia. Data were obtained from institutional electronic medical records covering the period between January 2021 and December 2023. The study was reported in accordance with the STROBE guidelines for observational studies (21).

### Study population

Adult patients aged 18–80 years with a confirmed diagnosis of gastric cancer who underwent surgical treatment were included. The prehabilitation protocol consisted of a comprehensive multidisciplinary assessment conducted by a nutritionist, physiotherapist, surgeon, and nursing staff. A full nutritional evaluation was performed, including nutritional diagnosis according to established criteria. Functional status was assessed using the Short Physical Performance Battery (SPPB), the Medical Research Council (MRC) scale for muscle strength, and a frailty assessment.

Preoperative risk factors were evaluated, and patients received structured preoperative education regarding fasting recommendations and preoperative carbohydrate loading with maltodextrin.

An individualized exercise prescription was provided for ambulatory implementation during the 14-day preoperative period. Exercise intensity and type were tailored according to each patient’s baseline functional capacity and frailty status.

Patients received a 5-day course of oral isocaloric immunonutrition formula containing omega-3 fatty acids, glutamine, arginine, and nucleotides (Inmunex^®^ Plus, Megalabs, USA), for a total of 15 sachets. The dosage was calculated based on a requirement of 0.3 g of arginine per kilogram of body weight, without exceeding 21 g per day, with a mean administration of three sachets per day.

The night before surgery, patients received 100 g of maltodextrin diluted in 800 mL of water. Two hours before surgery, 50 g of maltodextrin were administered in 400 mL of water (12.5% concentration).

Patients who were clinically eligible for postoperative oral intake continued the same immunonutrition regimen for an additional 5 days after surgery.

Patients were excluded if they were pregnant, had autoimmune diseases, chronic kidney disease requiring dialysis, or a diagnosis of synchronous malignancy. Exclusion criteria also included patients requiring additional surgical procedures on other organs (hepatobiliary, renal, gynaecological, or oesophageal), a history of bariatric surgery, or incomplete institutional medical records.

All patients underwent radical gastrectomy with D2 lymphadenectomy by laparoscopy, following a standardised institutional technique consistent with current international guidelines. Advanced bipolar energy devices were used in all cases, and anastomoses were performed using mechanical stapling.

### Variables

Sociodemographic, anthropometric, and clinical variables were analysed, including medical history, postoperative complications, transfusion requirements or ICU stay, haemoglobin levels, perioperative oncological management (chemotherapy or radiotherapy), disease stage, surgical technique, lymph node dissection level, degree of surgical radicality achieved, and intraoperative blood loss.

Nutritional status was assessed according to the Global Leadership Initiative on Malnutrition (GLIM) criteria (22). Sarcopenia was diagnosed based on handgrip strength using a Jamar Plus + 12–0604 digital dynamometer (cut-off: men < 27 kg; women < 16 kg). Sarcopenia severity was determined using the Short Physical Performance Battery (SPPB), following the updated recommendations of the European Working Group on Sarcopenia in Older People (EWGSOP2) (23).

The primary outcomes were in-hospital mortality and admission to the intensive care unit (ICU). Complications were classified according to the Clavien–Dindo scale, which stratifies postoperative complications based on the intervention required (medical management, procedures with or without anaesthesia, or death) (24).

Secondary outcomes included the frequency of infectious complications, postoperative bleeding, and the need for blood product transfusion.

### Data sources and bias control

Data were obtained from the institutional nutrition support and ERAS programme databases. A standardised instrument was used for data collection.

To minimise bias, investigators received prior training, and data quality control was ensured through random verification of 10% of the records. Before statistical analysis, consistency of the final database was verified.

### Statistical analysis

The distribution of quantitative variables was assessed using the Shapiro–Wilk test. Variables with normal distribution were presented as mean ± standard deviation (SD), while non-normally distributed data were expressed as median and interquartile range (IQR). Categorical variables were reported as absolute and relative frequencies (%).

Exploratory comparisons between groups were conducted using Pearson’s chi-squared test or Fisher’s exact test for categorical variables, and Student’s t-test or Mann–Whitney U test for quantitative variables, according to data distribution.

### Ethical considerations

The nutritional intervention was implemented as part of the institutional care protocol prior to the study. The study was approved by the Research Ethics Committee of Fundación Universitaria Sanitas (CEIFUS 2725-23). Given the retrospective nature of the research, the requirement for individual informed consent was waived.

## Results

**Fig. 1:**
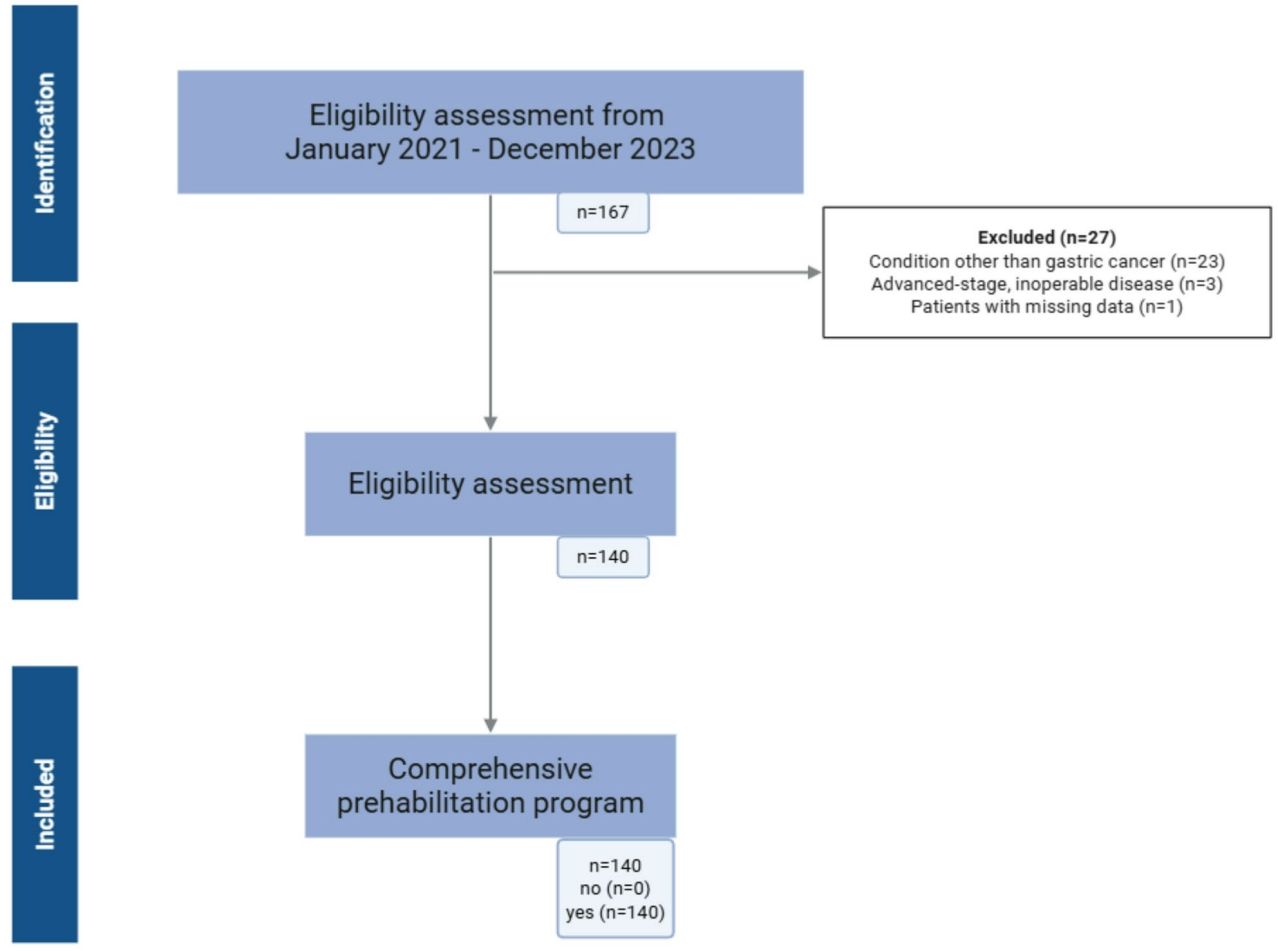
Flow diagram illustrating patient selection, inclusion, and analysis process in the study

A total of 140 patients diagnosed with gastric cancer were included in the analysis (Fig. 1). The mean age was 60.9 ± 13.3 years, with a predominance of male patients (60%). The most frequent comorbidities were arterial hypertension (31.4%) and type 2 diabetes mellitus (19.3%).

Regarding anthropometric characteristics, 48.6% of patients had a body mass index (BMI) within the normal range. However, according to the GLIM criteria, 74% of patients presented some degree of malnutrition, with 38% classified as moderate and 36% as severe malnutrition. Regarding anthropometric characteristics, 48.6% of patients had a normal BMI and only 6.4% were classified as underweight. However, malnutrition according to the GLIM criteria was identified in 74% of the cohort (38% moderate and 36% severe). This discrepancy can be explained by the fact that, within the GLIM framework, low BMI is only one of the three phenotypic criteria. The diagnosis can also be established based on reduced muscle mass or significant unintentional weight loss. Among the 102 patients diagnosed with malnutrition according to GLIM, only 9 presented with low BMI, whereas 47 had reduced muscle mass and 46 had moderate or severe weight loss.

In terms of tumour staging, 63.6% of the cohort were classified as stages IA–IIB according to the TNM system. All patients achieved negative surgical margins (R0), and 95.7% underwent D2 lymphadenectomy.

The majority (69.3%) received neoadjuvant treatment, predominantly chemotherapy regimens (Table 1). Most surgical procedures consisted of gastrectomies without associated interventions. Additional procedures such as splenectomy, oesophagectomy, pancreatectomy, or hemicolectomy were performed in fewer than 3% of patients.

**Table 1:** Demographic, clinical, and nutritional characteristics of the study population

Variable	Total (*n* = 140)
Mean age, years (SD)	60.9 ± 13.3
Sex, n (%)	
Male	84 (60.0)
Female	56 (40.0)
Body mass index, n (%)	
Underweight (< 18.5)	9 (6.4)
Normal (18.5–24.9)	68 (48.6)
Overweight (25–29.9)	45 (32.1)
Obesity (≥ 30)	18 (12.9)
Comorbidities, n (%)	
Hypertension	44 (31.4)
Type 2 diabetes mellitus	27 (19.3)
Chronic kidney disease	4 (2.9)
Mean haemoglobin, g/dL (SD)	12.7 ±
Nutritional diagnosis (GLIM criteria), n (%)	
No malnutrition	34 (25.0)
Moderate malnutrition	53 (38.0)
Severe malnutrition	49 (36.0)
TNM staging	
Stages IA–IIB	89 (63.6)
Stages IIIA–IV	51 (36.4)
R0 surgical margin	140 (100.0)
D2 lymphadenectomy	134 (95.7)
Neoadjuvant treatment,	
Chemotherapy	97 (69.3)
Radiotherapy	3 (2.0)

Postoperative complications occurred in 23.6% of patients. The most frequent complication was surgical site infection (17.1%), followed by postoperative bleeding (3.6%). A total of 7.1% of patients required intensive care unit (ICU) admission, and 2.1% received red blood cell transfusions.

According to the Clavien–Dindo classification, 16.4% of complications were grade II or higher, including 4.3% classified as grade V (mortality).

**Fig. 2:**
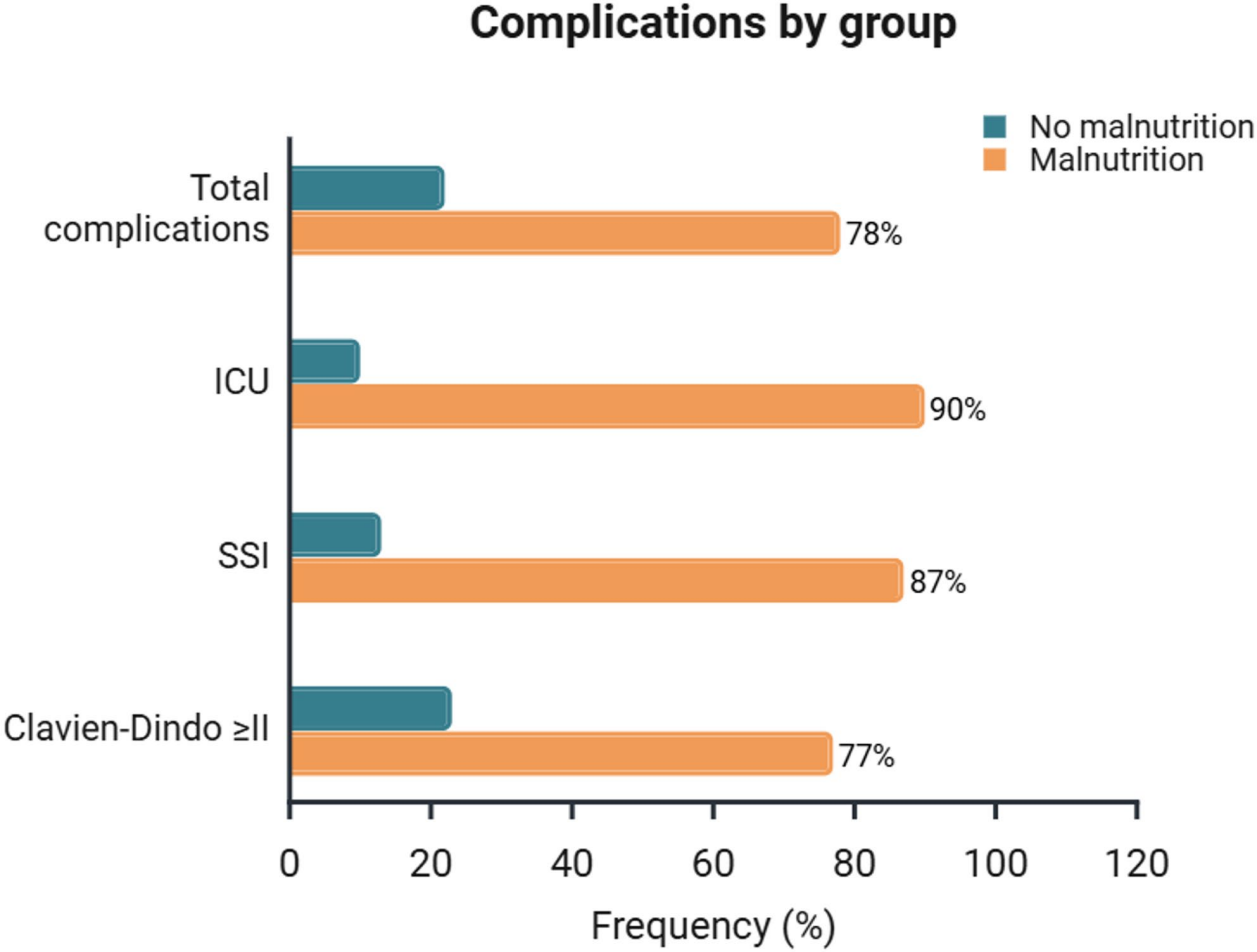
Frequency of complications (overall, ICU admission, surgical site infection, and those classified as Clavien-Dindo grade ≥ II), stratified by nutritional status. Malnutrition defined according to GLIM criteria

In the bivariate analysis, a low body mass index (BMI) was initially associated only with ICU admission. However, when BMI was analysed alongside malnutrition status (GLIM criteria), malnourished patients accounted for 78% of all complications, 90% of ICU admissions, 87% of surgical site infections, and 77% of Clavien–Dindo grade II or higher complications (Fig. 2).

No statistically significant associations were found between postoperative outcomes and age, sex, comorbidities, tumour stage, or neoadjuvant treatment.

## Discussion

Prehabilitation aims to modify factors most closely associated with adverse clinical outcomes, and its integration into gastric cancer management protocols has become increasingly common in recent years (6). In this cohort of 140 patients with gastric cancer, the overall postoperative complication rate was 23.6%, with surgical site infection (17.1%) being the most frequent, consistent with previous reports.

Although most patients had a BMI within the normal or overweight range, this parameter alone does not accurately represent the nutritional status of surgical candidates. Therefore, the GLIM criteria were applied to establish the diagnosis of malnutrition (25). These criteria have been validated as a reliable tool for the identification of nutritional risk and prediction of adverse postoperative outcomes in patients undergoing gastric cancer surgery (26).

Consistent with the current literature, the findings of this study support the relevance of preoperative nutritional assessment and optimisation as a core component of the prehabilitation process. This integrated approach addresses modifiable factors —such as malnutrition, sarcopenia, and reduced functional capacity— that have a direct impact on postoperative morbidity, functional recovery, and overall survival.

The present study reinforces the evidence supporting multidimensional prehabilitation programmes (nutritional, physical, and psychological) in oncological surgical patients and underscores the importance of their routine incorporation into enhanced recovery pathways within high-complexity institutions.

Physical exercise is a cornerstone of prehabilitation. Several authors have emphasised that assessing, training, and improving functional capacity prior to surgery leads to reduced postoperative decline and faster recovery (27). In 2024, Shen et al. demonstrated that prehabilitation —combining nutritional interventions, exercise, and psychological support— reduced postoperative complications by 23% (RR = 0.78; 95% CI: 0.66–0.93) (19). Similarly, previous studies reported a 30% reduction in major complications among frail patients undergoing abdominal surgery following multimodal prehabilitation programmes (28).

The nutritional component plays a pivotal role in optimising patients with gastric cancer. These individuals are particularly prone to malnutrition and frailty, conditions strongly associated with adverse surgical outcomes (29). A 2024 meta-analysis in patients undergoing major abdominal surgery revealed that individualised nutritional protocols aiming for an adequate protein intake (1.2–1.9 g/kg/day), often complemented with post-exercise leucine or immunonutrient supplementation, were linked to fewer complications and shorter hospital stays (30).

In our cohort, patients received multidisciplinary metabolic support, including the perioperative administration of immunomodulatory formulas enriched with omega-3 fatty acids, arginine, and glutamine. Perioperative enteral immunonutrition has been shown to improve inflammatory markers, shorten hospital stay, and decrease postoperative complications (31, 32).

The overarching goal of the prehabilitation programme was to enhance overall patient functionality through structured physical therapy and immunonutritional supplementation, promoting muscle mass gain and improved nutritional status —reflected in the parameters defined by the GLIM criteria (21). In our study, patients classified as malnourished consistently exhibited worse clinical outcomes, including higher rates of surgical site infection, ICU admission, and Clavien–Dindo grade II or higher complications (Fig. 2).

Our cohort presented common comorbidities such as hypertension (31.4%) and diabetes mellitus (19.3%), with 63.6% in early TNM stages and negative surgical margins (R0) in all cases. A D2 lymphadenectomy was performed in 95.7%, and neoadjuvant therapy was administered in 69.3%, aligning with international guidelines. The overall complication rate (23.6%) and postoperative mortality (4.3%) were comparable to previous international reports (33), where rates between 20% and 40% have been described depending on tumour stage, nutritional status, and comorbidities.

Although evidence on prehabilitation in gastric cancer remains emerging, recent studies demonstrate that structured multimodal programmes can significantly reduce postoperative complications (from 50% in the control group to 28% in the intervention group; *p* = 0.036) (34). Likewise, other authors have reported improved functional capacity (VO₂peak) and a trend toward reduced morbidity in oesophagogastric surgery (35).

Generalizability may be limited because this experience reflects implementation in a tertiary centre with specific multidisciplinary resources; feasibility and uptake may differ in non-tertiary settings where staffing, infrastructure, and follow-up capacity are more constrained.

Our findings underscore the importance of preoperative nutritional status as a determinant of postoperative outcomes, supporting multidimensional prehabilitation as an effective strategy to optimise surgical recovery in patients with gastric cancer. However, given the descriptive and hypothesis-generating nature of this study, these observations should be interpreted as exploratory and warrant confirmation in larger analytic cohorts.

## Conclusions

In this cohort of gastric cancer patients undergoing prehabilitation, the postoperative complication rate was 23.6% and mortality reached 4.3%, consistent with international data. Malnutrition, as defined by GLIM criteria, was consistently associated with worse postoperative outcomes, including higher rates of surgical site infection, ICU admission, and major complications.

These findings highlight the importance of incorporating prehabilitation as a core component of perioperative management in gastric cancer surgery. Optimising nutritional and functional status prior to surgery represents a safe, feasible, and potentially effective strategy to reduce complications, enhance recovery, and improve clinical outcomes in this high-risk population.

### Strengths and limitations

This study was conducted in a single high-complexity referral centre, ensuring uniformity in definitions, surgical procedures, and outcome assessment, thereby minimising variability and enhancing internal consistency. Moreover, it reflects the real-world implementation of a structured and comprehensive prehabilitation programme, adding practical relevance to its findings. Importantly, it represents the first local study in this field, contributing context-specific evidence and establishing a benchmark for future prospective and multicentre investigations.

Nevertheless, several limitations should be acknowledged. First, its retrospective design precludes causal inference and carries an inherent risk of information and selection bias. The bivariate analyses were exploratory and intended for hypothesis generation, not for establishing causal relationships. Second, being a single-centre cohort, the generalisability of the results to other healthcare settings is limited. Third, outcomes with low event frequency (e.g., bleeding, mortality, transfusion) reduced the statistical power and widened confidence intervals. Finally, the lack of comparable national data restricts external validation and underscores the need for prospective, multicentre studies with standardised GLIM-based nutritional assessment and clearly defined prehabilitation components.

## Data Availability

The data that support the findings of this study are available from Clínica Colombia. Restrictions apply to the availability of these data, which were used under authorization for the current study and are therefore not publicly available due to institutional policies and patient confidentiality. The data are, however, available from the corresponding author upon reasonable request and with permission of Clínica Colombia.
